# Effect of Applicator Brush Use on Color Change, Hydrogen Peroxide Penetration, and Gel Consumption in In-Office Bleaching: A Systematic Review and Meta-Analysis

**DOI:** 10.3390/dj14090533

**Published:** 2026-08-24

**Authors:** Samille Biasi Miranda, Rosalia Maux de Carvalho Rodrigues, Caroline de Farias Charamba Leal, Fernanda Campos, Hugo Rafael de Souza Cavalcante, Priscylla Gonçalves Correia Leite de Marcelos, Marcos Antonio Japiassú Resende Montes, Rodrigo Barros Esteves Lins

**Affiliations:** 1 Department of Dental Materials, Faculty of Dentistry, University of Pernambuco, Recife 50100-130, PE, Brazil; samille.biasi@upe.br (S.B.M.); caroline.charamba@upe.br (C.d.F.C.L.); marcos.japiassu@upe.br (M.A.J.R.M.); 2 School of Dentistry, Federal University of Alagoas, Maceio 57072-900, AL, Brazil; rosalia.rodrigues@foufal.ufal.br (R.M.d.C.R.); priscylla.marcelos@foufal.ufal.br (P.G.C.L.d.M.); 3 School of Dentistry, State University of Paraíba, Araruna 58429-500, PB, Brazil; fernanda.campos@servidor.uepb.edu.br

**Keywords:** dental bleaching, hydrogen peroxide, tooth bleaching, in-office bleaching, systematic review

## Abstract

**Background**: In-office dental bleaching is a common aesthetic procedure, and different application methods may influence laboratory and clinical outcomes. Objectives: This systematic review and meta-analysis evaluated the effects of applicator brush use compared with conventional application without a brush during in-office hydrogen peroxide bleaching. **Methods**: The review followed PRISMA guidelines and was registered in PROSPERO (CRD42023491451). Electronic searches were conducted in PubMed, Cochrane Library, LILACS, and OpenGrey for studies published until April 2026. Clinical and in vitro studies comparing bleaching gel application with and without an applicator brush were included. Outcomes included hydrogen peroxide concentration in the pulp chamber, bleaching gel consumption, color change, and tooth sensitivity. Risk of bias was assessed using RoB 2 for the randomized clinical trial and the Sarkis-Onofre tool for in vitro studies. Meta-analyses were performed using RevMan, and certainty of evidence was assessed using the GRADE approach. **Results**: Of 10,260 records identified, four studies met the eligibility criteria, including one randomized clinical trial and three in vitro studies. Quantitative synthesis was based exclusively on the three in vitro studies. The randomized clinical trial showed low risk of bias, whereas the in vitro studies presented methodological limitations. Meta-analyses showed no statistically significant differences between applicator brush and conventional application methods for hydrogen peroxide concentration in the pulp chamber or bleaching gel consumption, although pooled estimates showed a tendency toward lower values with applicator brush use. No significant differences were observed in color change outcomes assessed by ΔEab, ΔE00, or ΔWID (*p* > 0.05). Heterogeneity ranged from 0% to 99%, and the certainty of evidence for all outcomes was rated as very low. **Conclusions**: Based on predominantly laboratory evidence of very low certainty, applicator brush use may be associated with lower hydrogen peroxide concentration in the pulp chamber and reduced bleaching gel consumption; however, these findings should be interpreted cautiously, and the clinical relevance of this application method remains uncertain.

## 1. Introduction

Dental whitening is a widely sought procedure among those wishing to improve the appearance of their smile, addressing aesthetic problems associated with tooth discoloration [1]. As a conservative and widely used procedure, dental whitening has become part of routine dental practice, improving tooth appearance and patients’ aesthetic satisfaction [2].

This clinical procedure can be performed in the office by the dentist through the application of a high-concentration whitening gel directly onto the dental surface for 30 to 60 min per session, or through the at-home supervised technique, where a custom tray is made for the patient to place a low-concentration whitening gel under the professional’s guidance, carrying out the procedure at home for 30 min to 2 h daily, for a period of 2 to 6 weeks [3].

The main substance used in the in-office technique is hydrogen peroxide (HP), which has the ability to penetrate enamel and dentin through the diffusion of its byproducts that dissociate, releasing free radicals capable of oxidizing the molecules responsible for pigmentation. However, despite this technique promoting faster and satisfactory whitening, the diffusion of hydrogen peroxide toward the pulp chamber has been associated with the occurrence of dental sensitivity, characterized by mild to intense spontaneous pain within the first 48 h after the procedure [3].

The amount of whitening gel can also influence the development of dental sensitivity. According to Fick’s Second Law, larger volumes of hydrogen peroxide favor greater diffusion through dental tissues [4]. In this sense, an increase in the amount of gel is associated with a greater penetration of peroxide into the pulp chamber and a more intense inflammatory response, without necessarily resulting in greater whitening efficacy [4,5].

Thus, applying a thin and uniform layer of bleaching gel is important to avoid unnecessary material excess, and the use of an applicator brush may facilitate better control of gel distribution on the tooth surface [6]. In this context, whitening gels in auto-mixing syringes with applicator brush tips have been developed, with the main goal of providing better control and uniformity in gel application [6]. Therefore, applicator brush tips have been proposed as a technical modification to improve control and uniformity during gel application [7]. However, whether this approach effectively influences hydrogen peroxide diffusion, gel consumption, and clinical outcomes remains uncertain.

Although applicator brush tips have been introduced as an alternative method for controlling bleaching gel application, evidence regarding their effects on bleaching-related outcomes is limited and has not been systematically synthesized. Therefore, evaluating this application method may help clarify whether this technical modification provides measurable benefits or only represents a change in application protocol.

Therefore, the objective of this systematic review with meta-analysis was to evaluate the effects of applicator brush use during in-office hydrogen peroxide dental bleaching on bleaching-related outcomes compared with application without an applicator brush, based on the available experimental and clinical evidence. The null hypotheses were: (1) there would be no difference in hydrogen peroxide concentration in the pulp chamber between application methods; (2) there would be no difference in the amount of bleaching gel used between groups; and (3) there would be no difference in color change outcomes between groups.

## 2. Materials and Methods

### 2.1. Registration and Protocol

This systematic review was reported in accordance with the Preferred Reporting Items for Systematic Reviews and Meta-Analyses (PRISMA) (Supplementary Material Table S1) [8], and its protocol was registered in the International Prospective Register of Systematic Reviews database (PROSPERO-CRD42023491451).

### 2.2. Eligibility Criteria

The inclusion criteria were based on the Population-Intervention-Comparison-Outcomes-Study Design (PICOS) strategy: P (Population)—patients with vital permanent teeth/human or bovine teeth; I (Intervention)—in-office whitening gel with applicator brush a; C (Comparison)—in-office whitening gel without applicator brush; O (Outcome)—hydrogen peroxide concentration in the pulp chamber, amount of whitening gel used, and color change; S (Study Design)—In vitro and clinical studies. Thus, the research question was: “Does the use of an applicator brush in the in-office dental whitening technique promote better clinical performance?”

The following inclusion criteria were adopted: In vitro, in situ, randomized clinical trials, non-randomized clinical trials, studies that evaluated different methods of whitening gel application, and studies that performed in-office whitening.

Exclusion criteria were: narrative, systematic, integrative, and scoping reviews, letters to the editor, conference abstracts, studies that only evaluated one method of whitening gel application, and studies that performed supervised at-home whitening.

### 2.3. Search Strategy

The electronic search was conducted in the following databases: PubMed/MEDLINE, Cochrane Library, Latin American and Caribbean Literature in Health Sciences (LILACS), and Open-Grey Literature. Two researchers (S.B.M. and R.M.d.C.R.) conducted the search using a combination of specific Medical Subject Headings (MeSH), Descriptors in Health Sciences (DeCS), and keywords, combined using the Boolean operators AND and OR to identify all relevant studies, as shown in Table 1. The references of the included articles were manually tracked to identify additional eligible studies. No filter was applied for publication year or language.

### 2.4. Article Selection Process

The articles retrieved from the scientific databases were transferred to an online software (Rayyan, Qatar Computing Research Institute) [9]. For initial screening, titles and abstracts were considered, and duplicates were removed. The studies considered duplicates were only those related to the article title and year of publication. All the retrieved studies were selected for title and abstract reading by two researchers (S.B.M. and R.M.d.C.R). Based on the eligibility criteria, a full-text reading was conducted, and a third reviewer (M.A.J.R.) analyzed the inclusion and selection processes of the other two reviewers. Any discrepancies in defining a study between the reviewers were resolved through discussion.

If the study was not found in full text or did not present complete data to determine its inclusion, the corresponding authors of the respective study were contacted via email in order to obtain the necessary information to then define its inclusion or exclusion from this systematic review.

The article selection process was presented as a flowchart. The Mendeley software ver 2.141.0 (Elsevier, Mendeley) was used for reference management. The Kappa Score index [10] assessed the level of agreement between the evaluators for study inclusion.

After defining the eligible studies, key data were extracted, such as author name, publication year and location, study type, study objective, sample group, whitening gels used, description of the control and treatment groups, outcomes evaluated, and respective results.

### 2.5. Data Extraction and Collection Process

The data were manually collected from the included articles by a single reviewer (S.B.M.) and confirmed by a second reviewer (R.M.d.C.R). The data from the studies were tabulated and analyzed in a standardized Excel spreadsheet (Microsoft, Redmond, WA, USA). The variables collected from the studies included author name, publication year and location, study type, study objective, sample group, whitening gels used, description of the control and treatment groups, outcomes evaluated, and respective results.

### 2.6. Risk of Bias Assessment

Two independent reviewers (S.B.M. and R.M.d.C.R.) independently assessed the risk of bias of the randomized clinical trial using the Cochrane Risk of Bias 2 (RoB 2) tool(22 August 2019 version) [11]. The RoB 2 tool evaluates five domains: (1) bias arising from the randomization process; (2) bias due to deviations from intended interventions; (3) bias due to missing outcome data; (4) bias in measurement of the outcome; and (5) bias in selection of the reported result. Risk-of-bias judgments were performed for each outcome and each domain was classified as “Low risk of bias,” “Some concerns,” or “High risk of bias,” according to the RoB 2 signaling questions and algorithm. An overall risk-of-bias judgment for each outcome was then generated following the RoB 2 guidance.

For in vitro studies, the risk of bias was assessed using the modified tool proposed by Sarkis-Onofre et al. [12], which was specifically developed for the methodological assessment of laboratory-based studies and evaluates domains relevant to in vitro research. The evaluated domains were: (1) randomization of teeth; (2) caries-free teeth; (3) standardization of enamel or dentin surface samples; (4) sample size; (5) blinding of the examiner; (6) sample size calculation; and (7) reporting of complete outcome data. If the authors reported a parameter, the study was marked with “Y” (yes) for that specific parameter. If the information was not available, the study was marked with “N” (no). A study that reported one to three “Y” items was classified as high risk of bias, four or five “Y” items as medium risk, and six or seven “Y” items as low risk.

### 2.7. Meta-Analysis

The included in vitro studies that provided data regarding the outcomes of interest (hydrogen peroxide concentration in the pulp chamber, amount of whitening gel used, and whitening efficacy in different parameters) were analyzed using RevMan 5.3 software (Review Manager v. 5, The Cochrane Collaboration, Copenhagen, Denmark). Five meta-analyses were conducted: (1) concentration of 35% hydrogen peroxide in the pulp chamber; (2) amount of 35% hydrogen peroxide bleaching gel used; and color change assessed by (3) ΔEab, (4) ΔE00, and (5) ΔWID following bleaching with 35% hydrogen peroxide.

The randomized clinical trial was not included in the quantitative synthesis because its study design and outcomes were not comparable with those of the laboratory investigations. In the study by Centenaro et al., which evaluated multiple commercial bleaching products containing 35% hydrogen peroxide, only one comparison was included in each meta-analysis to avoid double-counting data from the same experimental sample. The comparison selected for the quantitative synthesis was DSP White Clinic 35% Calcium, as it showed greater methodological comparability with the other included studies, considering hydrogen peroxide concentration, bleaching protocol, and commercial characteristics of the bleaching product. This selection criterion was not prespecified in the review protocol and was defined during the data synthesis phase to minimize unit-of-analysis errors and improve comparability among studies.

For all meta-analyses, mean values, standard deviations, and sample sizes reported in the included studies were used to calculate the mean differences (MDs) and 95% confidence intervals (CIs) using a random-effects model. Statistical heterogeneity was assessed using the I^2^ statistic.

### 2.8. Assessment of Evidence Certainty

The results found in the risk of bias and meta-analysis were crucial for evaluating the certainty of the evidence using the Grading of Recommendations Assessment, Development and Evaluation (GRADE) classification [13], accessible at http://www.gradeworkinggroup.org/, accessed on 10 April 2026. The certainty of evidence was assessed based on the following domains: risk of bias, inconsistency, indirectness, imprecision, and publication bias. Therefore, it was classified as moderate, low, or very low evidence in cases of serious or very serious issues. Additionally, if there was a large or very large effect size, the quality of the evidence could improve. In this sense, the certainty of evidence may range from very low to high. On the other hand, non-randomized studies are initially considered low evidence and low quality, but may be downgraded if there are serious concerns in the five categories evaluated and described above.

## 3. Results

### 3.1. Study Selection

The electronic search was conducted in April 2026 and retrieved a total of 10,260 records: PubMed (n = 5735), LILACS (n = 1224), and Cochrane Library (n = 3297), in addition to four unique records identified through handsearching. After duplicate removal, 8615 records were screened based on titles and abstracts. Of these, 8602 records were excluded because they did not meet the eligibility criteria. Thirteen articles were retrieved for full-text assessment, of which nine were excluded for predefined reasons. Finally, four studies were included in the qualitative synthesis. Of these, three in vitro studies were included in the quantitative synthesis (meta-analysis).

Of the 13 full-text articles assessed, 9 were excluded. The reasons for exclusion were as follows: three studies compared different concentrations of bleaching gel without varying the applicator tip; one study compared the same experimental bleaching gel using the same applicator tip; one study included a negative control; and four studies were excluded because the Pola Office Plus bleaching gel was not applied using the applicator brush tip, preventing comparison between brush and non-brush application methods.

**Figure 1:**
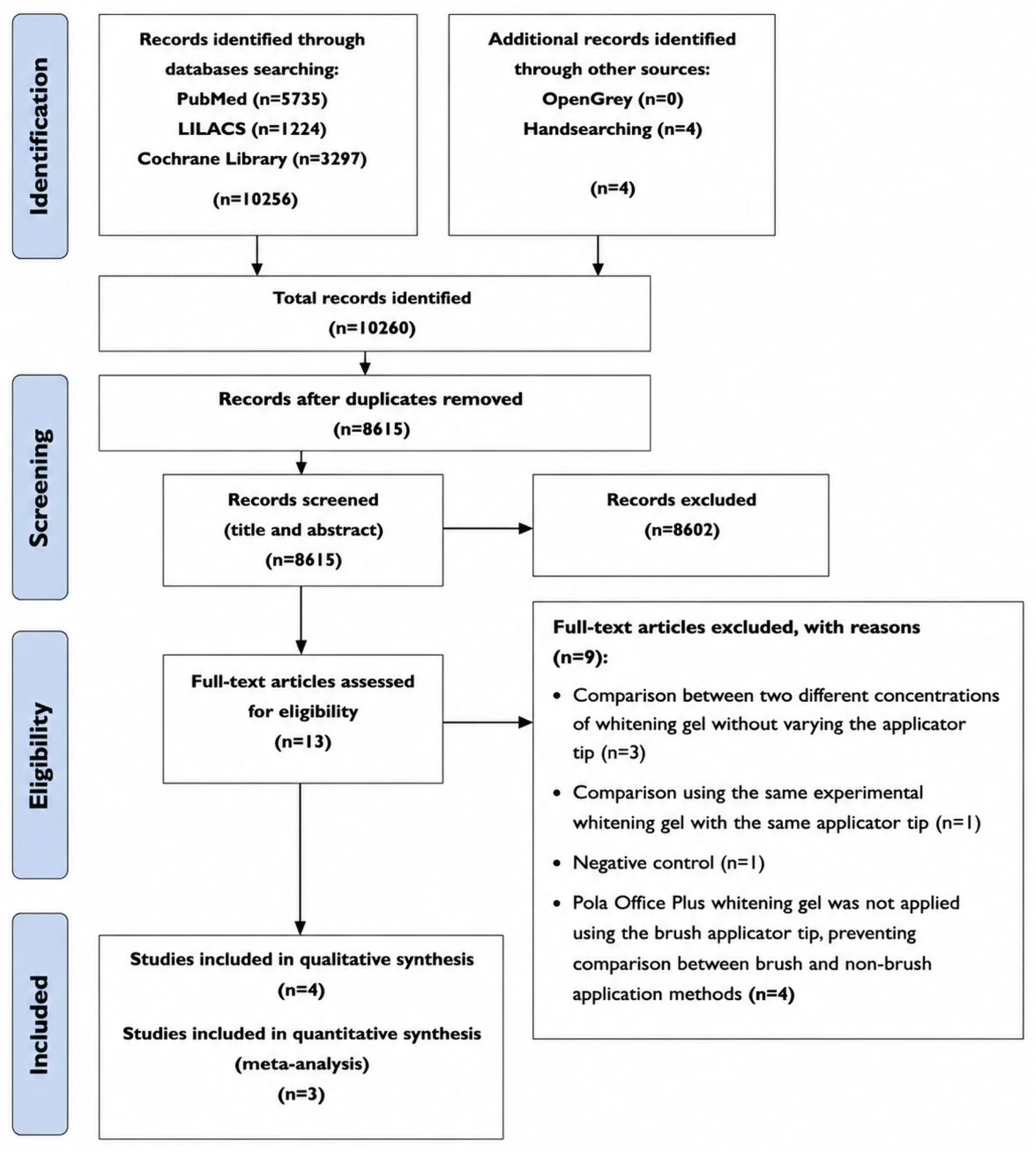
Flowchart of articles included in the systematic review.

As a result, four studies met the eligibility criteria and were included in the systematic review. Of these, three in vitro studies were eligible for quantitative synthesis (meta-analysis), whereas one randomized clinical trial was included only in the qualitative synthesis because its design and outcomes were not comparable with those of the laboratory studies (Figure 1).

The inter-examiner agreement for study selection was almost perfect (κ = 0.83), indicating strong consistency between reviewers.

### 3.2. Characteristics of Included Studies

Table 2 presents the main characteristics of the four included studies, comprising one randomized clinical trial (RCT) [14] and three in vitro studies [6,15,16], published between 2022 and 2024.

Sample sizes in the included studies ranged from 40 to 104 specimens in the laboratory studies and included 60 participants in the clinical trial [6,14,15,16]. The in vitro studies used extracted human premolars with standardized dimensions [6,15,16], whereas the clinical study included adolescents aged 12–16 years undergoing in-office bleaching treatment [14].

The bleaching protocols evaluated hydrogen peroxide concentrations ranging from 6% to 40% [6,14,15,16]. One in vitro study investigated both low- and high-concentration formulations (6% and 35%) [15], whereas the remaining studies evaluated commercially available bleaching products containing 6%, 35%, 37.5%, 38%, or 40% hydrogen peroxide [6,14,16]. A common methodological feature among all included studies was the comparison between applicator tips with and without a brush, aiming to evaluate the influence of gel distribution during bleaching procedures [6,14,15,16].

Regarding the evaluated outcomes, three in vitro studies assessed hydrogen peroxide concentration in the pulp chamber and the amount of bleaching gel used during application [6,15,16]. Color change was evaluated in all studies using instrumental methods based primarily on ΔEab, ΔE00, and ΔWID calculations [6,14,15,16]. Additionally, the clinical trial assessed the risk and intensity of tooth sensitivity associated with the bleaching procedures [14].

Overall, studies consistently demonstrated greater hydrogen peroxide diffusion into the pulp chamber and higher bleaching gel consumption when applicator tips without a brush were used [6,15,16]. In contrast, brush-containing applicators promoted more controlled gel distribution and reduced material consumption [6,15,16]. Regarding whitening effectiveness, both application methods produced clinically relevant color changes. Although some in vitro comparisons reported slightly higher color change values for groups treated without a brush [6,15], no statistically significant differences between application methods were observed in the quantitative synthesis.

Although all studies employed objective methods to evaluate bleaching outcomes, differences in substrate type, bleaching products, hydrogen peroxide concentrations, and assessment protocols may limit direct comparisons among studies [6,14,15,16]. Therefore, further standardized investigations are needed to strengthen the evidence regarding the clinical implications of using brush-containing applicator tips during in-office bleaching procedures.

### 3.3. Risk of Bias Assessment

**Figure 2:**
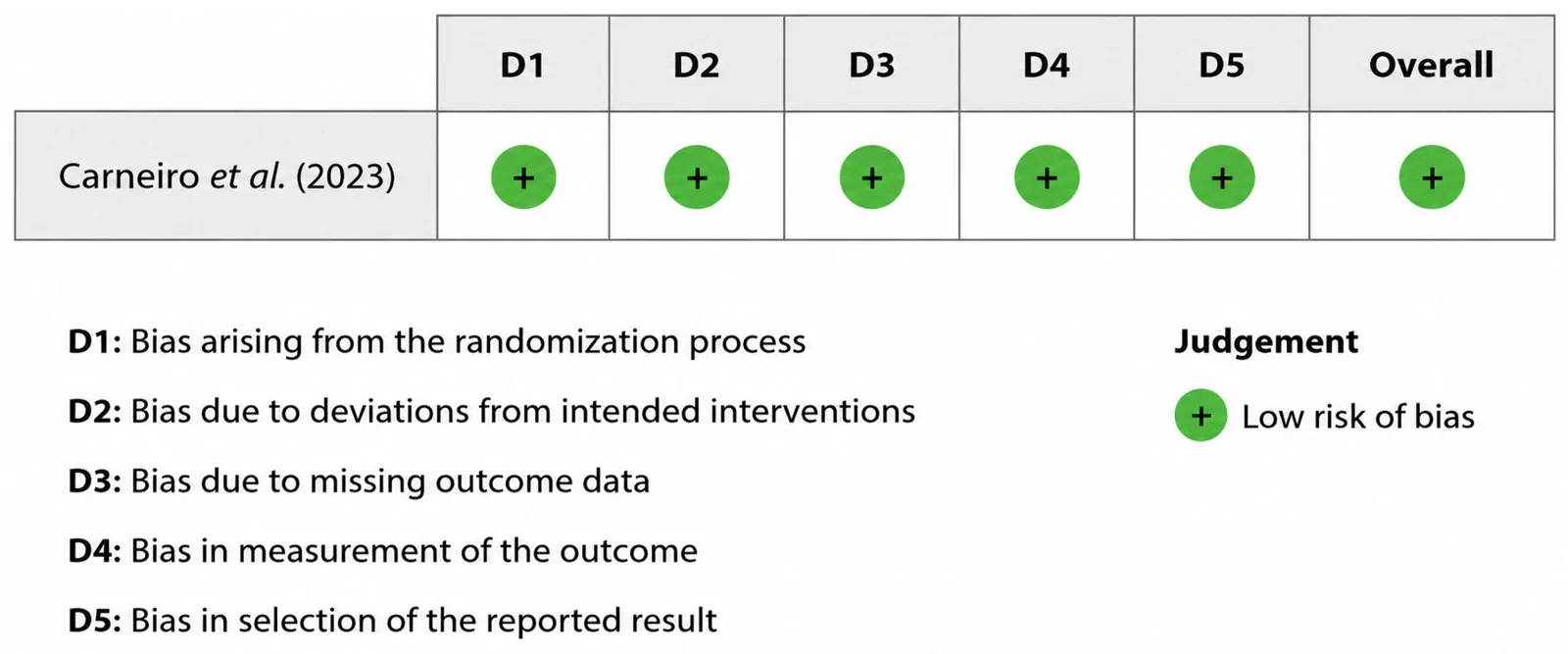
Risk of bias analysis of randomized clinical study [14].

The randomized clinical trial by Carneiro et al. (2023) [14] was assessed using the Cochrane RoB 2 tool. The risk of bias was evaluated for the relevant outcomes across the five RoB 2 domains (D1–D5). All domains were judged as low risk of bias, resulting in an overall judgment of low risk of bias for the assessed outcomes (Figure 2). These findings indicate adequate methodological quality. However, because only one randomized clinical trial met the eligibility criteria, the available clinical evidence remains limited and should be interpreted with caution.

In contrast, the three in vitro studies [6,15,16] were evaluated using the Sarkis-Onofre tool, revealing some variability across the assessed domains. In general, the studies demonstrated adequate tooth randomization, use of caries-free teeth, sample standardization, and complete reporting of results. However, recurring limitations included the lack of examiner blinding in all studies and the absence of sample size calculation in one study, which may introduce bias and affect the reliability of the findings (Table 3). All included in vitro studies were classified as having a moderate risk of bias, reflecting acceptable but not optimal methodological quality. These results should be considered when interpreting the outcomes and assessing the certainty of the evidence.

### 3.4. Meta-Analysis

Only data from the included in vitro studies [6,15,16] were eligible for quantitative synthesis. The randomized clinical trial [14] was not included in the meta-analysis because its outcomes and study design were not comparable with those of the laboratory investigations. Therefore, five separate meta-analyses were performed, including hydrogen peroxide concentration in the pulp chamber, bleaching gel consumption, and color change outcomes assessed by ΔWID, ΔE00, and ΔEab.

**Figure 3:**
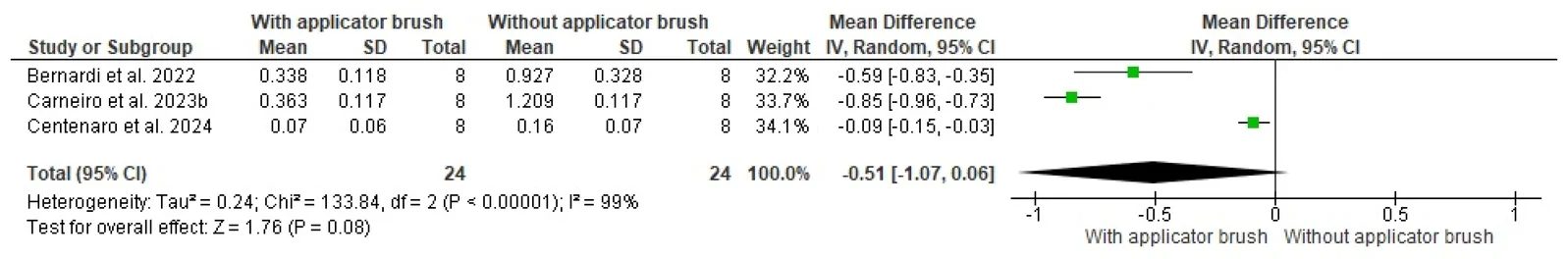
Meta-analysis evaluating bleaching gel (35% hydrogen peroxide) concentration in the pulp chamber obtained from in vitro studies [6,15,16].

The first meta-analysis (Figure 3) evaluated the concentration of hydrogen peroxide in the pulp chamber after bleaching procedures. The pooled analysis showed no statistically significant difference between applicator brush and conventional application methods (MD = −0.51; 95% CI: −1.07 to 0.06; *p* = 0.08). Although the effect estimate favored the use of the applicator brush, indicating lower hydrogen peroxide penetration into the pulp chamber, substantial heterogeneity was observed among studies (I^2^ = 99%).

**Figure 4:**
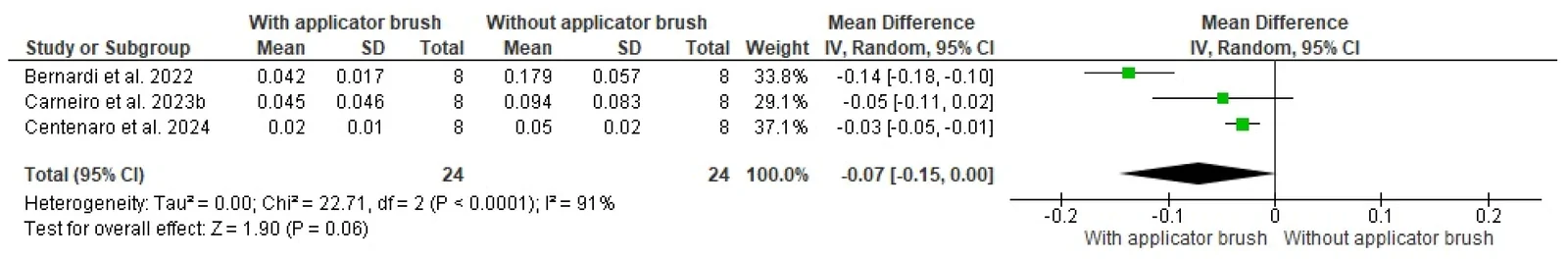
Meta-analysis evaluating bleaching gel (35% hydrogen peroxide) consumption obtained from in vitro studies [6,15,16].

The second meta-analysis (Figure 4) assessed the amount of bleaching gel used during application. The pooled estimate demonstrated no statistically significant difference between groups (MD = −0.07; 95% CI: −0.15 to 0.00; *p* = 0.06). The direction of the effect favored applicator brush use, suggesting lower gel consumption; however, considerable heterogeneity was detected among studies (I^2^ = 91%).

**Figure 5:**
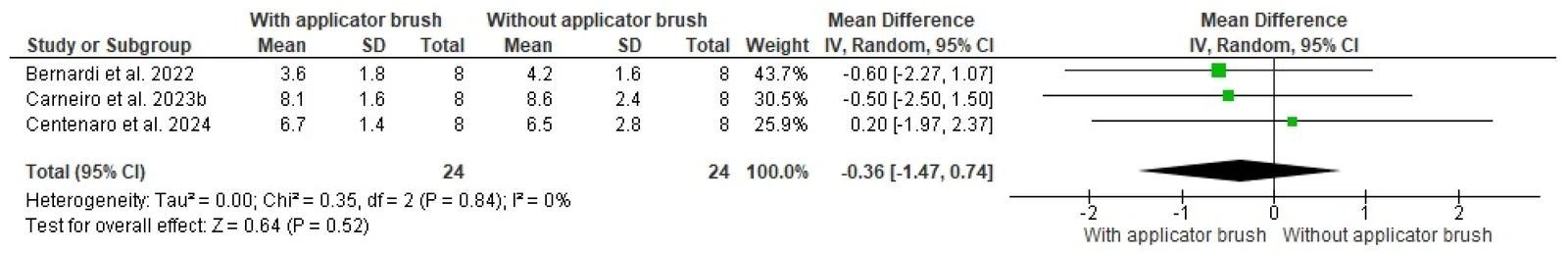
Meta-analysis evaluating ΔEab color change after bleaching with 35% hydrogen peroxide obtained from in vitro studies [6,15,16].

The third meta-analysis (Figure 5) evaluated color change using the CIELAB parameter (ΔEab). Similarly, no significant difference was observed between applicator brush and conventional application methods (MD = −0.36; 95% CI: −1.47 to 0.74; *p* = 0.52), with no heterogeneity detected (I^2^ = 0%).

**Figure 6:**
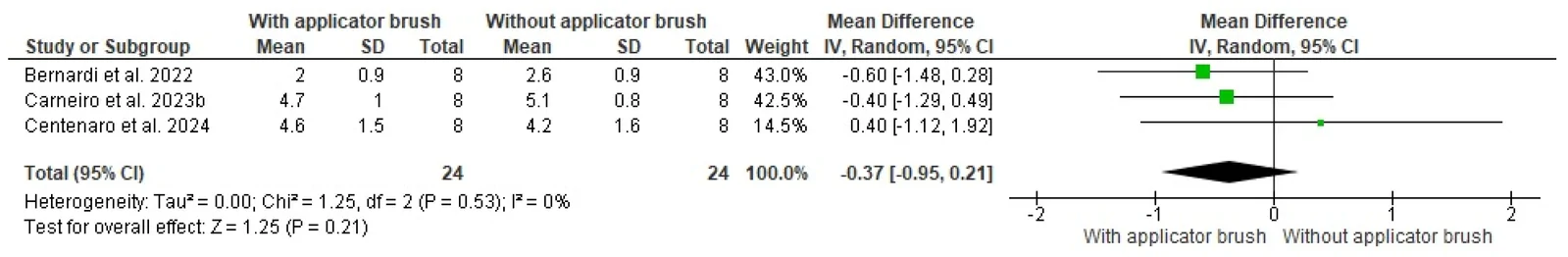
Meta-analysis evaluating ΔE00 color change after bleaching with 35% hydrogen peroxide obtained from in vitro studies [6,15,16].

The fourth meta-analysis (Figure 6) assessed color change using the CIEDE2000 parameter (ΔE00). The pooled analysis showed no statistically significant difference between groups (MD = −0.37; 95% CI: −0.95 to 0.21; *p* = 0.21), with no observed heterogeneity (I^2^ = 0%).

**Figure 7:**
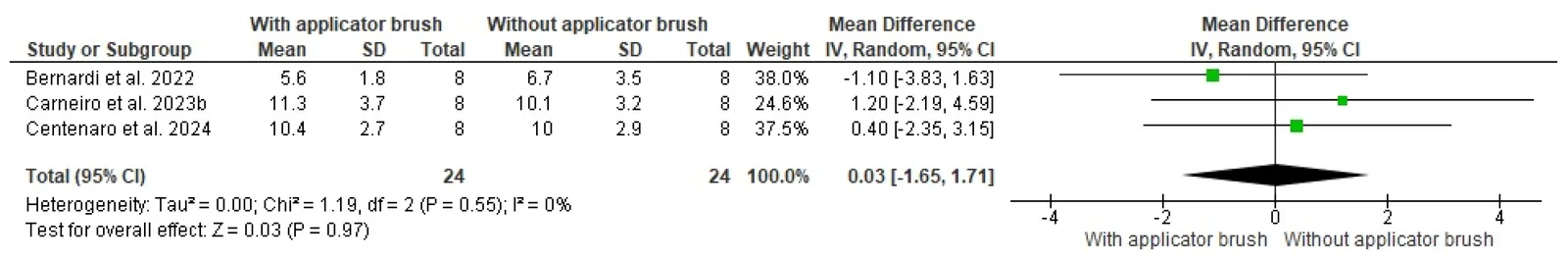
Meta-analysis evaluating ΔWID color change after bleaching with 35% hydrogen peroxide obtained from in vitro studies [6,15,16].

The fifth meta-analysis (Figure 7) evaluated whitening efficacy using the ΔWID parameter. No statistically significant difference was observed between applicator brush and conventional application methods (MD = 0.03; 95% CI: −1.65 to 1.71; *p* = 0.97), with no heterogeneity among studies (I^2^ = 0%).

Overall, the quantitative synthesis showed that applicator brush use did not significantly influence whitening efficacy, as assessed by ΔEab, ΔE00, and ΔWID. Regarding hydrogen peroxide concentration in the pulp chamber and bleaching gel consumption, the pooled estimates showed a tendency toward lower values with applicator brush use; however, these findings did not reach statistical significance and should be interpreted cautiously due to the substantial heterogeneity observed and the very low certainty of evidence.

### 3.5. Certainty of Evidence Assessment

Table 4 presents the certainty of evidence for the concentration of 35% hydrogen peroxide in the pulp chamber and the amount of 35% hydrogen peroxide gel used (n = 3 studies for each outcome). The certainty of evidence was rated as very low for both outcomes. The evidence was downgraded due to serious risk of bias, very serious inconsistency, serious indirectness, and serious imprecision. The risk of bias was mainly attributed to methodological limitations in the included in vitro studies, including lack of blinding and absence of sample size calculation. Inconsistency was considered very serious due to substantial variability in effect estimates and methodological differences among the included studies. Indirectness was rated as serious because the evidence was derived exclusively from in vitro studies, limiting the applicability of the findings to clinical conditions. Imprecision was also considered serious due to small sample sizes and the limited number of available comparisons. No additional considerations were identified.

Table 5 presents the certainty of evidence for the color change outcomes. For color change assessed by ΔEab, ΔE00, and ΔWID following bleaching with 35% hydrogen peroxide (n = 3 in vitro studies), the certainty of evidence was rated as very low. The evidence was downgraded due to serious risk of bias, serious indirectness, and serious imprecision. Indirectness was considered serious because all quantitative evidence was derived from in vitro studies, limiting the direct applicability of the findings to clinical practice. Inconsistency was not considered serious, as the pooled meta-analysis showed no statistical heterogeneity (I^2^ = 0%), indicating consistent results across the included studies.

## 4. Discussion

In-office dental bleaching is the procedure of choice for most professionals when patients seek to improve the aesthetics of their smile, due to its high success rate, faster results, and the fact that it does not require patient cooperation, as is the case with at-home techniques [17]. To provide a rapid whitening effect, in-office bleaching uses high concentrations of hydrogen peroxide (HP); however, a significant side effect is dental sensitivity, caused by the greater diffusion of HP into the dental pulp, leading to an inflammatory reaction in the tissue [18]. This issue may be influenced by the amount of gel applied on the enamel surface, as larger gel volumes have been associated with increased peroxide availability and greater peroxide penetration into dental tissues in experimental models. To reduce the amount of product applied and potentially limit hydrogen peroxide diffusion into the pulp chamber, whitening gels in self-mixing syringes with applicator brush tips have been proposed as an alternative application method [7].

Through this systematic review with meta-analysis, which primarily evaluated laboratory outcomes and included limited clinical evidence regarding the use or non-use of applicator brush tips in in-office bleaching, the null hypotheses were not rejected. No statistically significant differences were found in color change outcomes between groups with and without applicator brush use. Similarly, no statistically significant differences were detected in hydrogen peroxide concentration in the pulp chamber or bleaching gel consumption; however, a numerical tendency toward lower peroxide concentrations and reduced gel consumption was observed when the brush was employed. Therefore, these findings should be interpreted as mechanistic laboratory evidence rather than direct evidence of clinical benefit.

It is important to note that, regardless of the bleaching gel evaluated, he included studies generally showed a similar direction of effect, with a tendency toward lower hydrogen peroxide concentration in the pulp chamber and reduced gel consumption when an applicator brush was used during in-office bleaching [6,15,16]. These laboratory findings suggest that applicator brushes may provide greater control over gel application and potentially reduce material consumption. However, whether the observed reductions in hydrogen peroxide penetration and gel consumption translate into clinically meaningful outcomes remains to be determined.

A plausible explanation is that the use of an applicator brush may result in the application of a smaller amount of bleaching gel on the enamel surface, thereby reducing the total amount of hydrogen peroxide available for diffusion. Considering that the amount of bleaching gel applied may affect peroxide availability [4,19], and that hydrogen peroxide diffusion through dental tissues is influenced by peroxide concentration and application conditions [20], a smaller gel volume may reduce peroxide diffusion toward the pulp chamber. This laboratory mechanism may help explain the tendency toward lower peroxide concentrations observed in the pulp chamber when using an applicator brush, while maintaining sufficient contact between the bleaching agent and the dental surface to produce measurable color change. However, confirmation of this mechanism under clinical conditions is still lacking.

Overall, the findings indicated a tendency toward lower hydrogen peroxide concentration in the pulp chamber and reduced gel consumption when using the applicator brush; however, these differences did not reach statistical significance and were accompanied by substantial heterogeneity among studies. This variability may be explained by methodological differences across the included investigations. First, different dental substrates were used, including human premolars and bovine incisors, which may differ in enamel and dentin structure and permeability and may influence hydrogen peroxide diffusion. Second, the studies evaluated different commercial bleaching systems and hydrogen peroxide concentrations (35%, 37.5%, 38%, and 40%), which may affect gel viscosity, peroxide release kinetics, and interactions with dental tissues.

Furthermore, variations in tooth thickness, application protocols, and experimental conditions may have contributed to the observed heterogeneity, which may limit direct comparability across studies and should be considered when interpreting the pooled results. Despite these methodological differences, most included studies showed a similar direction of effect, with lower peroxide penetration and reduced gel consumption when applicator brushes were used [6,15,16].

Regarding color change, the individual meta-analyses showed no statistically significant differences between groups for ΔEab, ΔE00, or ΔWID when 35% hydrogen peroxide was used. These findings indicate that, under the evaluated laboratory conditions, the use of an applicator brush did not influence whitening efficacy. However, additional well-designed clinical studies are needed to determine whether these findings are maintained under clinical conditions.

The randomized clinical trial included in this review [14] was not eligible for quantitative synthesis because it used a low-concentration bleaching gel and evaluated outcomes that were not directly comparable with those of the laboratory studies. Nevertheless, its findings showed that bleaching with 6% hydrogen peroxide produced similar objective (ΔEab, ΔE00, and ΔWID) and subjective (ΔSGU) color change outcomes regardless of whether an applicator brush tip was used. However, because this study used a low-concentration bleaching gel and an adolescent population, extrapolation of these findings to conventional high-concentration in-office protocols should be made with caution.

Regarding dental sensitivity, the available clinical evidence is limited to a single randomized clinical trial [14]. Although reduced hydrogen peroxide penetration into the pulp chamber may represent a plausible biological mechanism for decreasing pulpal irritation, this hypothesis has not yet been confirmed under clinical conditions. Therefore, the current evidence is insufficient to establish the influence of applicator brush use on tooth sensitivity.

The main limitations of this review include the small number of eligible studies, particularly randomized clinical trials, with only one clinical study meeting the inclusion criteria. Importantly, all pooled analyses were derived exclusively from in vitro studies; therefore, the quantitative findings should be interpreted as laboratory evidence and cannot be directly extrapolated to clinical practice. Additionally, methodological heterogeneity among the included laboratory studies, limited sample sizes, and differences in bleaching protocols contributed to uncertainty in the findings and restricted the possibility of conducting additional subgroup analyses. The small number of included studies also precluded a formal assessment of publication bias, and its potential influence cannot be excluded. For all evaluated outcomes, including hydrogen peroxide concentration in the pulp chamber, bleaching gel consumption, and color change, the certainty of evidence was rated as very low according to the GRADE approach, mainly due to concerns related to risk of bias, inconsistency, indirectness, and imprecision. Furthermore, the absence of sufficient clinical evidence prevented quantitative synthesis of clinically relevant outcomes, such as tooth sensitivity, limiting the external validity of the findings.

Additionally, because Centenaro et al. [14] evaluated multiple commercial bleaching products, only one comparison was selected for quantitative synthesis to avoid double-counting of experimental data. Although DSP White Clinic 35% Calcium was chosen based on methodological comparability with the other studies, the potential influence of this analytical decision on pooled estimates should be acknowledged. Finally, although a highly sensitive search strategy was adopted, the extensive use of synonyms and related terms may have increased the complexity of the retrieval process.

The findings of this review suggest that the use of an applicator brush may be associated with reduced hydrogen peroxide penetration into the pulp chamber and lower bleaching gel consumption, based primarily on laboratory evidence. These results provide mechanistic insights rather than definitive clinical recommendations. Therefore, no definitive conclusions regarding patient outcomes can currently be drawn. Moreover, because the available evidence is predominantly derived from in vitro studies and is of very low certainty, the clinical implications of these findings should be interpreted with caution. Further well-designed clinical studies are needed to determine the clinical relevance of applicator brush use, particularly regarding patient-centered outcomes such as tooth sensitivity.

## 5. Conclusions

This systematic review and meta-analysis found no evidence that the use of an applicator brush during in-office bleaching influences color change outcomes, as assessed by ΔEab, ΔE00, and ΔWID. The quantitative synthesis showed a tendency toward lower hydrogen peroxide concentration in the pulp chamber and reduced bleaching gel consumption with applicator brush use; however, these differences did not reach statistical significance. The findings should be interpreted cautiously because the certainty of evidence was rated as very low, and the available studies presented methodological limitations and substantial heterogeneity. Further well-designed clinical studies are needed to determine the clinical relevance of brush-tip applicator use in in-office bleaching, particularly regarding patient-centered outcomes such as tooth sensitivity.

## Figures and Tables

**Table 1:** Electronic search strategies.

Database	Search Strategy
PubMed/MEDLINE	((((((tooth[MeSH Terms]) OR (tooth[Title/Abstract])) OR (permanent dentition[Title/Abstract])) OR (permanent dentition[MeSH Terms])) OR (teeth[Title/Abstract])) AND (((((((((((dental bleaching[Title/Abstract]) OR (tooth bleaching[Title/Abstract])) OR (bleaching gel[Title/Abstract])) OR (bleaching agent[Title/Abstract])) OR (hydrogen peroxide[Title/Abstract])) OR (in office bleaching[Title/Abstract])) OR (application[Title/Abstract])) OR (applicator tip[Title/Abstract])) OR (tip with brush[Title/Abstract])) OR (mixing tip[Title/Abstract])) OR (applicator brush[Title/Abstract]))) AND (((((((((((((((((((Clinical Trial[Title/Abstract]) OR Randomized Controlled Trial [Title/Abstract]) OR Clinical Study[Title/Abstract]) OR Clinical Trials, Randomized [Title/Abstract]) OR Trials, Randomized Clinical[Title/Abstract]) OR Controlled Clinical Trials, Randomized [Title/Abstract]) OR clinical protocols[Title/Abstract])))) OR clinical investigation [Title/Abstract]) OR clinical reports[Title/Abstract]))) OR nonrandomized [Title/Abstract] OR nonrandomized [Title/Abstract]))) OR (Clinical Evaluation[Title/Abstract])) OR ((((((((((In vitro Techniques[MeSH Terms]) OR Laboratories[MeSH Terms]) OR In vitro Technique*[Title/Abstract]) OR Technique*, In vitro[Title/Abstract]) OR In vitro as Topic[Title/Abstract]) OR In vitro[Title/Abstract]) OR Intervention Study[Title/Abstract]) OR Longitudinal Study[Title/Abstract]) OR Laboratory[Title/Abstract]) OR (Investigation[Title/Abstract]) OR (In situ[Title/Abstract]) OR (Laboratories[Title/Abstract])))
LILACS	((mh:(tooth)) OR (tooth) OR (mh:(permanent dentition)) OR (permanent dentition) OR (teeth)) AND ((dental bleaching) OR (tooth bleaching) OR (bleaching gel) OR (bleaching agent) OR (hydrogen peroxide) OR (in office bleaching) OR (application) OR (applicator tip) OR (tip with brush) OR (mixing tip) OR (applicator brush)) AND ((Clinical Trial) OR (Randomized Controlled Trial) OR (Clinical Study) OR (Clinical Trials, Randomized) OR (Trials, Randomized Clinical) OR (Controlled Clinical Trials, Randomized) OR (clinical protocols) OR (clinical investigation) OR (clinical reports) OR (nonrandomized) OR (Clinical Evaluation) OR (mh:(In vitro Techniques)) OR (mh:(Laboratories)) OR (In vitro Technique*) OR (Technique*, In vitro) OR (In vitro as Topic) OR (In vitro) OR (Intervention Study) OR (Longitudinal Study) OR (Laboratory) OR (Investigation) OR (In situ) OR (Laboratories))
Cochrane Library	(“tooth” OR “teeth” OR “permanent dentition”) AND (“dental bleaching” OR “tooth bleaching” OR “bleaching gel” OR “bleaching agent” OR “hydrogen peroxide” OR “in office bleaching” OR “application” OR “applicator tip” OR “tip with brush” OR “mixing tip” OR “applicator brush”) AND (“randomized controlled trial” OR “clinical trial” OR “randomized clinical trial” OR “clinical study” OR “clinical trials, randomized” OR “controlled clinical trials, randomized” OR “clinical protocols” OR “clinical investigation” OR “clinical reports”)
OpenGray	(tooth OR teeth OR “permanent dentition”) AND (“dental bleaching” OR “tooth bleaching” OR “bleaching gel” OR “bleaching agent” OR “hydrogen peroxide” OR “in-office bleaching”) AND (“applicator brush” OR “applicator tip” OR “brush tip” OR “mixing tip” OR application)

**Table 2:** Characteristics of the included studies.

Author, Year	Bernardi et al. (2022) [6]	Carneiro et al. (2023) [14]	Carneiro et al. (2023) [15]	Centenaro et al. (2024) [16]
Study type	In vitro	RCT	In vitro	In vitro
Sample group	Upper premolars, ≥A2 with thickness between 2.5 and 3.5 mm (n = 40)	Patients aged 12–16 years (n = 60); Half-arches (n = 120); Upper canines/≥A2	Upper premolars, ≥A2 with thickness between 2.5 and 3.5mm (n = 40)	Upper human premolars (n = 104), distributed into 13 groups (n = 8/group).
Whitening gel	Whiteness HP Automixx 35% (FGM) and Pola Office Plus 37.5% (SDI)	Whiteness HP Automixx 6% (FGM)	Whiteness HP Automixx 6% (FGM) and Whiteness HP Automixx 35% (FGM)	SP White Clinic 35% Calcium (DSP); Nano White 35% (DMC); Total Blanc One-Step 35% (DFL); Whiteness HP Blue 35% (FGM); Potenza Bianco Pro SS 38% (PHS); Opalescence Xtra Boost 40% (Ultradent)
Groups	HP 35% with applicator brush (n = 8); HP 35% without applicator brush (n = 8); HP 37.5% with applicator brush (n = 8); HP 37.5% without applicator brush (n = 8)	Tip without brush (n = 60); Tip with brush (n = 60)	HP 6% with brush (n = 8); HP 6% without brush (n = 8); HP 35% with brush (n = 8); HP 35% without brush (n = 8)	DSP White Clinic 35% Calcium: without brush (n = 10) and with brush (n = 10); Nano White 35%: without brush (n = 10) and with brush (n = 10); Total Blanc One-Step 35%: without brush (n = 10) and with brush (n = 10); Whiteness HP Blue 35%: without brush (n = 10) and with brush (n = 10); Potenza Bianco Pro SS 38%: without brush (n = 10) and with brush (n = 10); Opalescence Xtra Boost 40%: without brush (n = 10) and with brush (n = 10)
Concentration of HP in the pulp chamber	Cary 50-UV-Vis spectrophotometer (µg/mL); HP 35% with brush = 0.338 ± 0.118 HP 35% without brush = 0.927 ± 0.328 HP 37.5% with brush = 0.229 ± 0.092 HP 37.5% without brush = 0.511 ± 0.156	NR	Cary 50-UV-Vis spectrophotometer (µg/mL); HP 6% with brush = 0.012 ± 0.006 HP 6% without brush = 0.039 ± 0.033 HP 35% with brush = 0.363 ± 0.117 HP 35% without brush = 1.209 ± 0.783	DSP White Clinic 35% Without brush = 0.88 ± 0.25 With brush = 0.32 ± 0.10 Nano White 35% Without brush = 0.91 ± 0.28 With brush = 0.34 ± 0.11 Total Blanc One-Step 35% Without brush = 0.79 ± 0.22 With brush = 0.29 ± 0.08 Whiteness HP Blue 35% Without brush = 0.83 ± 0.24 With brush = 0.30 ± 0.09 Potenza Bianco Pro SS 38% Without brush = 0.95 ± 0.27 With brush = 0.36 ± 0.12 Opalescence Xtra Boost 40% Without brush = 0.86 ± 0.26 With brush = 0.31 ± 0.10
Amount of gel used	Analytical precision digital scale (g): HP 35% with brush = 0.042 ± 0.017 HP 35% without brush = 0.179 ± 0.057 HP 37.5% with brush = 0.156 ± 0.051 HP 37.5% without brush = 0.324 ± 0.098	NR	Analytical precision digital scale (g): HP 6% with brush = 0.041 ± 0.027 HP 6% without brush = 0.088 ± 0.046 HP 35% with brush = 0.045 ± 0.026 HP 35% without brush = 0.094 ± 0.083	DSP White Clinic 35%: Without brush = 0.17 ± 0.05; With brush = 0.05 ± 0.02; Nano White 35%: Without brush = 0.18 ± 0.06; With brush = 0.05 ± 0.02; Total Blanc One-Step 35%: Without brush = 0.16 ± 0.05; With brush = 0.04 ± 0.02; Whiteness HP Blue 35%: Without brush = 0.18 ± 0.06; With brush = 0.05 ± 0.02; Potenza Bianco Pro SS 38%: Without brush = 0.19 ± 0.06; With brush = 0.06 ± 0.02; Opalescence Xtra Boost 40%: Without brush = 0.18 ± 0.05; With brush = 0.05 ± 0.02.
Color Evaluation	∆Eab/∆E00/∆Wid HP 35% with brush = 3.6 ± 1.8/2.0 ± 0.9/5.6 ± 1.8 HP 35% without brush = 4.2 ± 1.6/2.6 ± 0.9/6.7 ± 3.5 HP 37.5% with brush = 4.6 ± 2.2/2.8 ± 0.9/6.9 ± 1.8 HP 37.5% without brush = 5.7 ± 2.4/3.5 ± 1.2/7.1 ± 2.0	∆Eab/∆E00/∆Wid Tip without brush 1 week after = 6.0 ± 3.4/4.2 ± 2.6/5.5 ± 4.9 2 weeks after = 7.3 ± 3.2/4.9 ± 2.5/8.9 ± 6.8 3 weeks after = 8.6 ± 3.2/5.7 ± 2.4/9.5 ± 5.4 1 month after = 8.2 ± 2.8/5.5 ± 2.2/10.5 ± 6.3 Tip with brush 1 week after = 6.4 ± 4.4/4.5 ± 3.3/3.9 ± 5.9 2 weeks after = 7.3 ± 3.4/5.0 ± 2.7/7.0 ± 6.6 3 weeks after = 8.5 ± 3.6/5.6 ± 2.7/7.2 ± 5.8 1 month after = 8.5 ± 3.8/5.8 ± 2.9/8.9 ± 6.4	∆Eab/∆E00/∆Wid HP 6% with brush = 4.5 ± 0.9/2.8 ± 0.6/5.7 ± 1.5 HP 6% without brush = 6.7 ± 1.4/4.5 ± 1.1/8.2 ± 2.6 HP 35% with brush = 8.1 ± 1.6/4.7 ± 1.0/11.3 ± 3.7 HP 35% without brush = 8.6 ± 2.4/5.1 ± 0.8/10.1 ± 3.2	∆Eab/∆E00 DSP White Clinic 35%: Without brush = 8.40 ± 2.00/5.10 ± 1.00; With brush = 8.10 ± 1.80/4.90 ± 1.00; Nano White 35%: Without brush = 8.60 ± 2.10/5.30 ± 1.10; With brush = 8.30 ± 1.90/5.10 ± 1.10; Total Blanc One-Step 35%: Without brush = 8.20 ± 1.90/5.00 ± 1.00; With brush = 7.90 ± 1.80/4.80 ± 1.00; Whiteness HP Blue 35%: Without brush = 8.50 ± 2.00/5.20 ± 1.00; With brush = 8.20 ± 1.90/5.00 ± 1.10; Potenza Bianco Pro SS 38%: Without brush = 8.70 ± 2.20/5.40 ± 1.20; With brush = 8.40 ± 2.00/5.20 ± 1.10; Opalescence Xtra Boost 40%: Without brush = 8.50 ± 2.10/5.20 ± 1.10; With brush = 8.20 ± 1.90/5.00 ± 1.00.
Sensitivity test	NR	EVA 0–10 (Risk and Intensity of SD): Yes/No Tip without brush = 27/60 Tip with brush = 20/60	NR	NR

Legend: RCT: randomized clinical trial; EVA: analytical visual scale; PH: hydrogen peroxide; SD: dental sensitivity; NR: not reported.

**Table 3:** Assessment of the risk of bias of the studies included in this systematic review: (Y) yes or (N) no.

Study	Tooth Randomization	Care-Free Teeth	Standardization of Enamel/Dentin Surface Samples	Single-Operator HP Application	Examiner Blinding	Sample Size Calculation	Complete Results Data Report	Risk of Bias
Bernardi et al. (2022) [6]	Y	Y	Y	N	N	Y	Y	Medium
Carneiro et al. (2023) [15]	Y	Y	Y	Y	N	Y	Y	Medium
Centenaro et al. (2024) [16]	Y	Y	Y	Y	N	N	Y	Medium

**Table 4:** Certainty of evidence assessed using the GRADE approach for hydrogen peroxide concentration in the pulp chamber and bleaching gel consumption after 35% hydrogen peroxide bleaching.

Certainty Assessment—Whitening Gel (35% Hydrogen Peroxide)									
Parameter	No of Studies	Study Design	Risk of Bias	Inconsistency	Indirectness	Imprecision	Other Considerations	Important	Certainty
Hydrogen peroxide concentration in the pulp chamber after bleaching with 35% hydrogen peroxide	3	In vitro	Serious ^a^	Very Serious ^b^	Serious ^c^	Serious ^d^	None	-	⊕◯◯◯ Very Low
Bleaching gel consumption during in-office bleaching with 35% hydrogen peroxide	3	In vitro	Serious ^a^	Very Serious ^b^	Serious ^c^	Serious ^d^	None	-	⊕◯◯◯ Very Low

Classification of the certainty of evidence according to the GRADE Working Group. ⊕◯◯◯ Very low quality: There is great uncertainty regarding the estimate. ^a^ All included in vitro studies presented a moderate risk of bias, mainly due to the absence of examiner blinding and, in one study, the lack of sample size calculation. ^b^ Substantial heterogeneity was observed across studies regarding experimental design, application protocols, and outcome assessment. ^c^ Indirectness was judged as serious because all quantitative evidence was derived from in vitro studies, limiting the direct applicability of the findings to clinical practice. ^d^ The evidence was downgraded due to small sample sizes and the limited number of included studies.

**Table 5:** Certainty of evidence assessed using the GRADE tool—Color change.

Certainty Assessment—Color Change									
Parameter	No of Studies	Study Design	Risk of Bias	Inconsistency	Indirectness	Imprecision	Other Considerations	Important	Certainty
Color change (ΔEab) after bleaching with 35% hydrogen peroxide	3	In vitro	Serious ^a^	Not Serious ^b^	Serious ^c^	Serious ^d^	None	-	⊕◯◯◯ Very Low
Color change (ΔE00) after bleaching with 35% hydrogen peroxide	3	In vitro	Serious ^a^	Not Serious ^b^	Serious ^c^	Serious ^d^	None	-	⊕◯◯◯ Very Low
Color change (ΔWID) after bleaching with 35% hydrogen peroxide	3	In vitro	Serious ^a^	Not Serious ^b^	Serious ^c^	Serious ^d^	None	-	⊕◯◯◯ Very Low

^a^ Although the studies were classified as having moderate risk of bias, the certainty of evidence was downgraded due to methodological limitations, including lack of examiner blinding and absence of sample size calculation. ^b^ Inconsistency was not considered serious because the pooled meta-analysis showed no statistical heterogeneity (I^2^ = 0%), indicating consistent results across the included studies. ^c^ Indirectness was judged as serious because all quantitative evidence was derived from in vitro studies, limiting the direct applicability of the findings to clinical practice. ^d^ The evidence was downgraded due to small sample sizes and the limited number of included studies. ⊕◯◯◯ Very low quality: There is great uncertainty regarding the estimate.

## Data Availability

All the data are available within the manuscript.

## Supplementary Materials

The following supporting information can be downloaded at: https://www.mdpi.com/article/10.3390/dj14090533/s1, Table S1: Preferred Reporting Items for Systematic Reviews and Meta-Analyses (PRISMA) guidelines.
